# Gut microbiota-melatonin signaling axis in acute pancreatitis: Revealing the impact of gut health on pancreatic inflammation and disease severity in a case-control study

**DOI:** 10.1097/MD.0000000000038689

**Published:** 2024-07-12

**Authors:** Chao Li, Yangfen Wen, Qiwen Tong, Yi Peng, Dan Yu, Yisong Rao, Yuehong Zeng

**Affiliations:** a Department of General Surgery, Yiyang Central Hospital, Yiyang City, China.

**Keywords:** acute pancreatitis, biomarkers, disease severity, gut microbiota, inflammation, melatonin, prediction

## Abstract

Acute pancreatitis (AP), a severe inflammatory condition affecting the pancreas requires investigation into its predictors. Melatonin, a compound with anti-inflammatory and antioxidant properties, has shown promise in managing AP. Additionally, the gut microbiota, a community of microorganisms residing in the intestines has been linked to AP development. This study aims to explore the correlation between melatonin and gut microbiota in predicting AP severity. This study involved 199 participants, with 99 diagnosed with AP and 100 serving as healthy controls. The AP patients were categorized into 2 groups based on the severity of their condition: mild AP (MAP) and severe AP (SAP). Serum melatonin levels were measured on Days 1, 3, and 5 of hospitalization, and gut microbiota composition was examined via 16S rRNA gene sequencing. Other parameters were evaluated, such as the Acute Physiology and Chronic Health Evaluation (APACHE) score, Ranson, and Acute Gastrointestinal Injury (AGI) scores. Melatonin levels were significantly lower in subjects with severe AP compared those with mild AP (18.2 ng/mL vs 32.2 ng/mL, *P* = .001), and melatonin levels decreased significantly in patients with AP on Days 3 and 5. The study also revealed that individuals with AP exhibited a significantly altered gut microbiota composition compared to control individuals, with a lower Shannon index and higher Simpson index. The AUCs for Simpson index and F/B ratio were significantly higher than those for other biomarkers, indicating that these gut microbiota markers may also be useful for AP prediction. The study proposes that there is a relationship between melatonin levels and the dynamics of gut microbiota profiles in relation to the severity of AP. As a result, the severity of the disease can be assessed by assessing the levels of serum melatonin and gut microbiota profiles.

## 1. Introduction

Acute pancreatitis (AP), a severe inflammatory disorder of the pancreas described by local pancreatic inflammation and systemic inflammatory response, poses a considerable burden on healthcare systems worldwide.^[1]^ While the exact mechanisms underlying AP pathogenesis remain incompletely understood, recent studies have highlighted the critical interplay between the gut microbiota, melatonin signaling, and AP severity.^[2–4]^

The gut microbiota, a diverse community of microorganisms inhabiting the intestines, plays a vital role in continuing intestinal homeostasis, immune regulation, and metabolic processes.^[5]^ Dysbiosis, characterized by an altered composition and diversity of the gut microbiota, has been identified as a prevalent feature of several chronic disorders, including AP.^[4,6,7]^ Dysbiotic microbiota have been implicated in inducing inflammation, impairing gut barrier function, and disrupting nutrient metabolism, all of which contribute to the development and progression of AP.^[8]^ Recent research indicates that the gut microbiota may influence melatonin signaling, potentially affecting the inflammatory response and disease course in AP.^[9]^ Studies have demonstrated that gut microbiota can modulate melatonin production, metabolism, and receptor expression, thereby influencing the bioavailability and efficacy of melatonin in the body.^[9,10]^

Melatonin, a hormone primarily produced by the pineal gland, exhibits potent anti-inflammatory and antioxidant properties. Its levels are altered in AP patients, suggesting a potential role in the disease’s pathophysiology.^[11]^ Melatonin exerts its effects through various mechanisms, including modulation of immune cells, inhibition of inflammatory signaling pathways, and scavenging of free radicals.^[10]^ To the best of our knowledge, no empirical investigations have examined the relationship between melatonin concentrations, gut microbiota profiles, and gut metabolite levels in patients with acute pancreatitis.^[11]^ The precise mechanisms by which melatonin may influence the development or progression of AP remain to be elucidated. A growing body of evidence suggests that melatonin plays a multifaceted role in the gut, circadian rhythms, regulating microbial metabolism, and intestinal mucosal immune cells.^[9,12]^ Melatonin receptors are ubiquitously expressed throughout the gastrointestinal tract, and melatonin levels in the gut are significantly higher, ranging from 400-fold to 10-100-fold more concentrated, compared to levels in the pineal gland or serum.^[13,14]^ Recent studies have demonstrated an inverse association between serum melatonin levels and the severity of acute pancreatitis.^[4,11,15]^ However, no studies have investigated the potential mediating role of the gut microbiota in this association.

Empirical evidence supports the idea that gut microbiota plays a central role in the pathogenesis of AP, influencing inflammation, gut permeability, glycemic control, lipid metabolism, insulin sensitivity, and host energy homeostasis.^[8]^ This study hypothesizes that gut microbiota may modulate the signaling effects of melatonin and consequently impact the severity of AP. The primary objective of this investigation was to examine the dynamic changes in plasma levels of endogenous melatonin during the first, third, and fifth days after AP onset in humans to assess its potential as a prognostic marker for AP severity and to predict severe AP (SAP). Additionally, the study aimed to evaluate the association between serum melatonin levels and the risk of AP. Furthermore, changes in gut metabolites (LPS, TMAO) and gut microbiota profiles were assessed between individuals with mild AP, severe AP, and healthy controls during the study period. By addressing these research questions, this study seeks to provide novel insights into the intricate interplay between gut microbiota and melatonin signaling in the pathogenesis of AP. These insights could potentially pave the way for the development of innovative therapeutic strategies for the prevention and treatment of this debilitating condition.

## 2. Materials and methods

### 2.1. Patients

To explore the potential influence of gut microbiota on the relationship between melatonin and AP, a robust case-control study was designed. The study included 99 AP patients and 100 healthy controls, all recruited from Yiyang Central Hospital in July 2021 and 2023 in the first 24 hours of admission to the hospital. Biliary causes accounted for the most cases (39%), followed by idiopathic (30%) and hyperlipidemic (20%). Other contributing factors included alcoholic and alcoholic and pancreatic duct obstruction (11%).

All participants provided written informed consent, and the study was conducted in accordance with the Declaration of Helsinki guidelines. Our study received approval from the local ethics committee of Yiyang Central Hospital (ethical code: 2020-020).

The study included patients who had not received any immunosuppressive drugs, antibiotics, or blood transfusions before enrollment. The APACHE II and Ranson scores, which are measures of the severity of acute pancreatitis, were 8 (range 0–19) and 2.5 (range 0–9), respectively. The patients were admitted to the gastroenterology department of our hospital between July 2020 and March 2023. The median age of the patients was 61.9 years (range 30–85 years). Organ failure was defined as a partial pressure of oxygen (PaO2) of less than 60 mm Hg at room air or the need for mechanical ventilation, systolic blood pressure of less than 90 mm Hg, or a serum creatinine level of greater than 2 mg/dL after rehydration or hemodialysis. According to the guidelines for the diagnosis and treatment of acute pancreatitis,^[16]^ 46 cases were classified as mild acute pancreatitis (MAP) and 53 cases were classified as severe acute pancreatitis (SAP).

The inclusion criteria for this study are patients with acute pancreatitis who have two of the following 3 characteristics: abdominal pain consistent with acute pancreatitis, serum amylase and/or lipase activity at least 3 times higher than the normal upper limit, and characteristic AP imaging changes on enhanced CT/MRI or abdominal ultrasound.

The exclusion criteria for this study are patients with organ transplantation, long-term use of immunosuppressive drugs, diseases that affect serum melatonin levels, combined tumors, abandoned treatment, incomplete information, or who are under the age of 18.

### 2.2. Severity of AP

The severity of intestinal dysfunction was categorized into 2 groups: non-intestinal failure group and intestinal failure group, based on the 2012 ESCCI/AGI classification criteria.^[17]^ AGI (grade I, II) was classified as non-intestinal failure group, while AGI (grade III, IV) was classified as intestinal failure group. Acute pancreatitis was graded as mild, moderate, or severe according to the 2012 Atlanta classification criteria. All patients with AP had their APACHEII score calculated within the first 24 hours of admission. The APACHEII score is a widely used tool for assessing the severity and prognosis of AP. It is based on 12 monitoring indicators and comprises an acute physiology score, an age index, and a chronic health index. Additionally, we calculated the Ranson’s scores using data obtained during the initial 48 hours after admission. Ranson’s criteria are an early scoring system used for predicting the severity of AP. It consists of a total of 11 parameters, out of which 5 factors are evaluated at the time of admission, and the remaining 6 factors are assessed during the subsequent 48 hours.

### 2.3. Medical history

To gather information on participants’ lifestyles, including alcohol consumption, physical activity, and dietary habits, we administered a validated questionnaire. Additionally, we collected medical histories to assess prior health conditions and potential risk factors for acute pancreatitis.

### 2.4. Blood samples

Blood samples were collected from the abdomen at 8:00 am on the first day of admission to measure serum melatonin levels. This timing was chosen to capture melatonin levels in the early morning, when they are naturally at their highest.

### 2.5. Serum melatonin levels

Three blood samples were collected in the morning after an overnight fast on consecutive days (Day 1, Day 3, and Day 5). Melatonin levels in these blood samples were measured using ELISA kits from Shanghai Mlbio Biotechnology Co. (ELISA Kit; Cat No. CK-bio -20626, China).

To assess the inflammatory response and pancreatic function, serum levels of C-reactive protein (CRP) and pancreatic amylase were measured using an automated analyzing platform. CRP is a marker of inflammation, while pancreatic amylase is an enzyme released from the pancreas during pancreatitis.

### 2.6. Lipopolysaccharide (LPS), cytokines, and TMAO

Serological levels of LPS, a component of bacterial cell walls, were measured to assess intestinal permeability and potential gut microbial dysbiosis. Additionally, levels of the cytokines interleukin-6 (IL-6) and tumor necrosis factor-alpha (TNF-α) were determined to gauge the severity of inflammation. Finally, trimethylamine N-oxide (TMAO), a gut microbial metabolite associated with cardiovascular disease, was measured.

### 2.7. Gut microbiota analysis

Stool samples were obtained in sterile containers within the first 24 hours of patients’ hospital admission, allowing for timely analysis and characterization. The DNA was extracted from these samples using the QIAamp Fast DNA Stool Mini Kit and confirmed through agarose gel electrophoresis. The hypervariable regions 3 and 4 of the 16S ribosomal RNA gene were amplified using specific primers, and paired-end sequencing was performed using the MiSeq platform. The alpha and beta diversity of the gut community were assessed using the Shannon and Inverse Simpson indices, respectively, and visualized using principal coordinate analysis plots based on unweighted UniFrac distance.^[18]^ The Firmicutes to Bacteroidetes (F/B) ratio was calculated to analyze the microbial composition. To quantify this ratio, we conducted real-time PCR with SYBR Green detection using the Rotor Gene 3000 instrument. Each reverse transcription PCR (RT-PCR) reaction mixture contained 10 mM of primers for both Firmicutes and Bacteroidetes, 5 µL of SYBR Green Master mix from Fermentase in Waltham, MA, USA, and 2 µL of DNA template, making a final reaction volume of 10 µL. To determine the F/B ratio, we obtained colony-forming unit (CFU) counts by using universal primers targeting the bacterial 16S rRNA gene along with specific primers for Firmicutes and Bacteroidetes.^[19]^

### 2.8. Statistical analysis

The data was analyzed using SPSS version 18 (IBM Corp, Released 2010, IBM SPSS Statistics for Windows, Version 19.0, Armonk, NY: IBM Corp) and STATA version 13 software (StataCorp, 2023, Stata 13 Base Reference Manual, College Station, TX: Stata Press). Significance was set at *P* values less than .05. Quantitative data was reported as mean ± standard deviation, whereas qualitative data was expressed as frequency (%). Binary logistic regression was used to assess the association between biomarkers and AP in the adjusted model. The receiver operating characteristic (ROC) analysis was performed to determine the best cutoff points for biomarkers, considering sensitivity and specificity. The ROC curve comparison tests were used to identify the best anthropometric indices for predicting AP. Among parametric, semi-parametric, and non-parametric approaches to estimate the area under the ROC curves, the parametric method was found to be least appropriate for analysis. The methods commonly used for establishing the “optimal” cut-point were the point on the ROC curve closest to (0,1) and the Youden index.

## 3. Results

The study included a total of 199 participants to investigate the relationship between serum melatonin and AP severity. The characteristics of the participants are presented in Table 1. There was a significant age difference between those with mild AP (MAP) and severe AP (SAP), with patients in the MAP group being significantly younger than those in the SAP group (53 years vs 64 years, *P* = .001). There was no difference in gender distribution between the 2 groups. The APACHEII, Ranson, and Acute Gastrointestinal Injury (AGI) scores at admission were significantly higher in the SAP group compared to the MAP group (APACHEII scores: 6.6 points vs 12.7 points, *P* < .001; Ranson scores: 3.4 points vs 6.38 points, *P* < .001). Three participants in the MAP group experienced organ failure, and no mortality occurred in this group, whereas, the SAP group experienced a higher incidence of organ failure, with 13 participants affected, and a mortality rate of 4 out of 53 (7.5%) was observed.

**Table 1:** Characteristics of patients with acute pancreatitis.

Variables	Mild (n = 46)	Severe (n = 53)	Total (n = 99)	*P* value*
Age (years)	53.54 ± 14.6	64.02 ± 8.46	61.9 ± 12.55	.001
Male	37 (37)	43 (43)	80	.156
Female	9 (9)	10 (10)	19	
BMI (kg/m^2^)	26.64 ± 3.07	26.66 ± 4.06	27.5 ± 3.9	.971
Alcohol intake n (%)	7 (15)	10 (18)	17	.049
APACHEII	6.6 ± 2.19	12.7 ± 2.8	9.94 ± 4.1	.001
Ranson	3.47 ± 1.4	6.38 ± 1.43	4.97 ± 1.99	.001
SOSF/MOSF	3 (6)	13 (24)	16	.001
Acute Gastrointestinal Injury (AGI)				
Grade I, II	44 (95)	46 (86)	90	.001
Grade III, IV	2 (5)	7 (14)	9	
Amylase (U/L)	1136.66 ± 260.1	1442.64 ± 464.07	1275.1 ± 496.1	.003

The values are presented as either mean ± standard deviation and number (%).

AGl = Acute Gastrointestinal Injury, APACHEII = Acute Physiology Chronic Health Evaluation II, BMI = body mass index (kg/m^2^), SOSF/MOSF = single/multiple organ systemic failure.

* *P* value were calculated using the independent *t* test.

The characteristics of both study groups are presented in Table 2. Participants in the case group had higher levels of fasting blood sugar, interleukin-6, CRP, and TNF compared to the control group. There was a marginal difference in systolic blood pressure between the 2 groups (*P* = .884), and there are significant differences in melatonin levels on day 1 between the cases and controls (47 vs 25, *P* < .000). However, while melatonin levels remained steady in the control group throughout Day 3 and Day 5 of sampling, there was a significant decrease in serum concentrations of melatonin on Days 3 and 5 among participants in the case group. The serum levels of melatonin were significantly lower in the SAP group compared to the MAP group in Day 5 (18.2 ng/mL vs 32.2 ng/mL, *P* = .001).

**Table 2:** Characteristics of participants with acute pancreatitis (cases) and matched control individuals.

Variables	Cases			*P* value*	Control (n = 100)	*P* value†
	Mild (n = 46)	Severe (n = 53)	Total (n = 99)			
Melatonin Day 1, pg/mL	47.65 ± 11.2	47.44 ± 11.89	47.6 ± 11.2	.845	25.33 ± 6.2	**<.000**
Melatonin Day 3, pg/mL	36.16 ± 6.17	28.01 ± 6.5	32.2 ± 12.2	**.002**	25.36 ± 6.17	**.001**
Melatonin Day 5, pg/mL	32.2 ± 7181	18.2 ± 7.4	24.74 ± 14.74	**.001**	22.2 ± 7.1	**.001**
FBS	116.1 ± 15.2	157.1 ± 14.2	130.1 ± 14.2	.437	111.1 ± 25.2	**.004**
SBP	127.2 ± 19.2	127.1 ± 26.2	127.2 ± 18.2	.984	126.1 ± 20.2	.884
DBP	78.5 ± 11.2	75.1 ± 8.04	81.1 ± 11.2	.324	85.1 ± 8.2	**.009**
IL-6	33.5 ± 9.97	35.1 ± 9.97	34.1 ± 9.97	.669	22.1 ± 3.97	**.001**
CRP	4.36 ± 1.7	7.5 ± 2.5	6.2 ± 3.3	.114	2.37 ± 1.63	**.001**
TNF	530.2 ± 141.3	610.2 ± 156.1	573.2 ± 145.1	.129	442.2 ± 182.2	**.001**

The values are presented as either mean ± standard deviation. Bold values denote statistical significance at the *P* < 0.05 level.

CRP = C-reactive protein, DBP = diastolic blood pressure, F/B = Firmicutes/Bacteroidetes Ratio, FBS = fasting blood sugar, IL-6 = interleukin-6, LPS = lipopolysaccharide, SBP = systolic blood pressure, TMAO = Trimethylamine N-oxide, TNF = tumor necrosis factor.

* *P* value were calculated using the independent *t* test.

† *P* trends were calculated using the one-way ANOVA test.

Table 3 displays characteristics related to gut microbiota profiles for participants with AP and control individuals. Notably, individuals with AP exhibited a significantly altered microbiota composition compared to the control group. Analysis of α-diversity revealed that the AP case group had a significantly lower diversity of microbiota, as evidenced by a lower Shannon index and higher Simpson index (*P* < .05), indicating that individuals with AP have a different gut microbial structure compared to control individuals. Additionally, analysis of TMAO and LPS levels revealed significantly higher values in the AP case group compared to the control group (TMAO: 55.5 ng/mL vs 23.3 ng/mL, *P* < .001; LPS: 0.59 ng/mL vs 0.23 ng/mL, *P* < .001).

**Table 3:** Characteristics of Gut Microbiota Profiles participants with acute pancreatitis (cases) and matched control individuals.

Variables	Cases			*P* value*	Control (n = 100)	*P* value†
	Mild (n = 46)	Severe (n = 53)	Total (n = 99)			
TMAO	53.6 ± 8.10	56.8 ± 13.2	55.6 ± 7.9	0.845	23.1 ± 3.13	**.001**
LPS	0.60 ± 0.23	0.59 ± 0.24	0.59 ± 0.23	0.957	0.26 ± 0.11	**.001**
Shannon diversity index	2.79 ± 1.1	2.89 ± 1.1	2.8 ± 1.1	0.673	3.6 ± 0.40	**.001**
Simpson diversity index	0.36 ± 0.11	0.44 ± 0.17	0.43 ± 0.22	0.615	0.43 ± 0.22	**.001**
Firmicutes/bacteroidetes (F/B) ratio	3.2 ± 1.2	3.4 ± 1.2	3.3 ± 1.3	0.614	1.9 ± 0.89	**.001**

The values are presented as either mean ± standard deviation. Bold values denote statistical significance at the *P* < 0.05 level.

F/B = Firmicutes/Bacteroidetes Ratio, LPS = Lipopolysaccharide, TMAO = Trimethylamine N-oxide.

* *P* value were calculated using the independent *t* test.

† *P* trends were calculated using the one-way ANOVA test.

In this study, we explored the correlation between AP and various biochemical variables, with AP serving as the dependent variable, while biochemical markers such as Melatonin on Days 1, 3, and 5, IL-6, CRP, TNF, TMAO, LPS, and Shannon index were considered as independent variables (independent predictors). Among all the biomarkers analyzed, Melatonin on Day 5 was found to have a significant association with an increased risk of APs (odds ratio: 0891, 95% confidence interval: 088–091, *P* < 001) (Table 4).

**Table 4:** Multiple-unadjusted odds ratios and 95% confidence intervals for Acute pancreatitis across biomarkers.†

Variables	OR (95% CI)	*P* *
Melatonin Day 1, pg/mL	00.975 (0.931–1.01)	.0260
Melatonin Day 3, pg/mL	0.994 (0.993–1.04)	.769
Melatonin Day 5, pg/mL	0891 (0.88–0.91)	<.001
IL-6	1.20 (0.98–1.20)	.666
CRP	1.24 (1.02–1.43)	.006
TNF	1.03 (1.02–1.04)	.161
TMAO	1.05 (1.02–1.07)	**.001**
LPS	0.98 (0.93–2.4)	.957
Shannon diversity index	0.19 (0.08–0.43)	**.001**
Simpson diversity index	1.57 (0.27–8.04)	.613
Firmicutes/bacteroidetes (F/B) ratio	1.69 (1.34–2.1)	**.001**

Bold values denote statistical significance at the *P* < 0.05 level.

CRP = C-reactive protein, F/B = firmicutes/bacteroidetes ratio, IL-6 = interleukin-6, LPS = = lipopolysaccharide, TMAO = Trimethylamine N-oxide, TNF = tumor necrosis factor.

* *P* based on linear regression and adjusted for age, sex, BMI.

† Analysis limited to case group.

**Figure 1.**
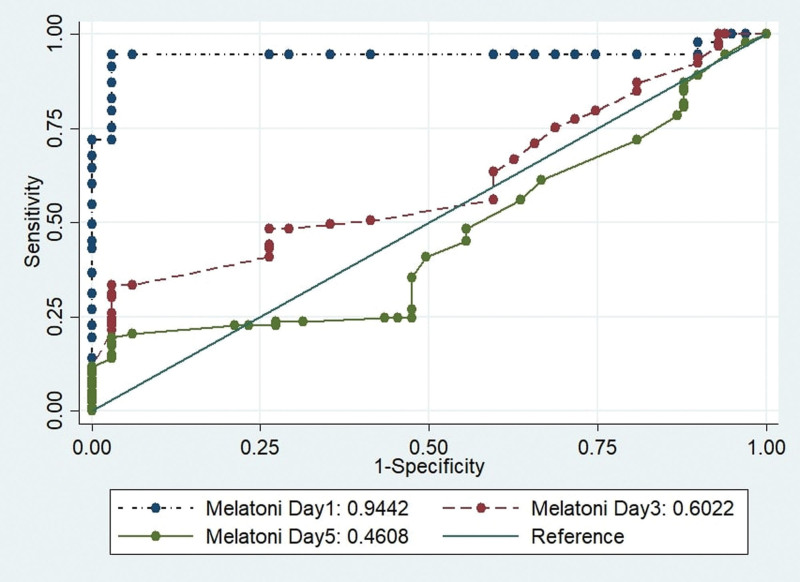
The AUC for melatonin on day 1, 3 and 5. AUC = area under the curve.

**Figure 2.**
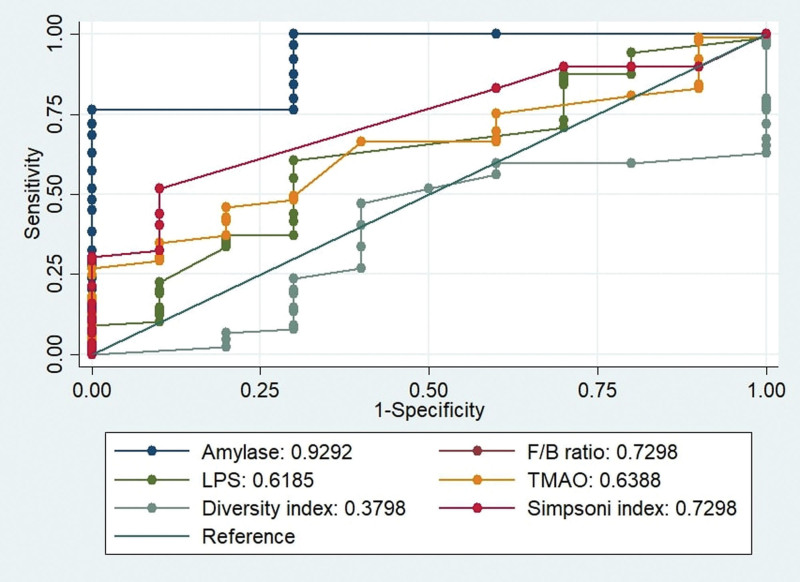
The AUCs and ROC-analysis results for characteristics related to gut microbiota profiles and AP. AUC = area under the curve, AP = acute pancreatitis, ROC = receiver operating characteristic.

The area under the curve (AUC) for melatonin on day 1 was significantly higher than on days 3 and 5, as shown in Figure 1. The AUC for melatonin on day 1 (AUC = 0.94, 95% CI = 0.91–0.98), was also greater than on days 3 and 5. The AUC for melatonin on day 3 was the lowest among all studied days (AUC = 0.60, 95% CI = 0.58–0.62). Similarly, Figure 2 displays the AUCs and receiver operating characteristic (ROC)-analysis results for characteristics related to gut microbiota profiles and AP. The AUCs for Simpson index and F/B ratio were significantly higher than those for other biomarkers, indicating that these biomarkers may be more predictive for AP than others. The ROC curve for Simpson index (AUC = 0.72, 95% CI = 0.70–0.74) was higher than the others for AP.

## 4. Discussion

The present study explored the relationship between serum melatonin and AP severity, as well as the association of melatonin and gut microbiota with AP. The findings revealed that melatonin levels were significantly higher in patients with severe AP compared to those with mild AP, suggesting a potential role of melatonin dysregulation in AP progression. Additionally, the study identified a significant decrease in melatonin levels on Days 3 and 5 among participants with AP, suggesting a dynamic pattern of melatonin changes during AP progression. These findings are consistent with previous studies which have reported decreased melatonin levels in AP patients.^[11,20]^ The study also suggests that melatonin may influence gut microbiota, as higher levels of melatonin were associated with alterations in microbial composition and like diversity of gut microbiota. The study highlights the importance of gut microbiota profiles, as Simpson index and F/B ratio were found to be more predictive for AP than other characteristics. These findings suggest that melatonin may have potential therapeutic benefits for AP patients, and further research is needed to explore the relationship between melatonin, gut microbiota, and AP severity.

The results of this study suggest that melatonin levels are significantly lower in patients with SAP compared to those with MAP patients on day 5 post-onset, but not at the initial stage of the disease. Furthermore, the study found that melatonin levels decreased significantly in patients with AP on days 3 and 5, while remaining steady in the control group, with melatonin levels possibly fluctuating throughout the course of AP. These fluctuations could be influenced by various factors, including the severity of inflammation, oxidative stress, and individual variations in melatonin production and metabolism.

Specifically, melatonin on day one was found to have higher predictive value for severe AP than melatonin on day 3 and 5. Additionally, the study employed ROC analysis to assess the predictive ability of melatonin and gut microbiota markers for AP. The AUC for melatonin on Day 1 was significantly higher than on Days 3 and 5, suggesting that melatonin levels on Day 1 may be the most predictive biomarker for AP. These findings are in line with a previous study by Jin et al (2019), which reported that melatonin levels were significantly higher in patients with SAP compared to healthy controls.^[11]^ However, the study by Belyaev et al did not investigate the temporal changes in melatonin levels in patients with AP.^[20]^ The findings of this study regarding the association between serum melatonin levels and the severity of AP are intriguing and warrant further exploration. It is important to note that the results contradict some previous studies that reported lower melatonin levels in AP patients. This discrepancy may be attributed to several factors, including variations in patient populations, sample collection timing, and the dynamic nature of melatonin levels during the course of AP.^[11,15,20]^ The mechanism by which melatonin is involved in the pathogenesis of AP is not fully understood. However, some studies have suggested that melatonin may have a protective effect against oxidative stress and inflammation, which are key factors in the development of AP.^[2,9,12]^ The study proposes that melatonin may protect cells from oxidative damage and reduce inflammation by stabilizing cell membranes, scavenging oxygen and nitrogen free radicals, and regulating cytokine production. The study suggests that patients with AP who have lower levels of melatonin are less likely to develop severe AP characterized by longer disease duration, systemic inflammatory response syndrome (SIRS) organ failure, and pancreatic necrosis. This finding supports previous research that has linked lower melatonin levels with higher AP severity.^[20,21]^

Recent research suggests that melatonin plays a significant role in regulating microbial metabolism as well as circadian rhythms and intestinal immune function.^[10]^ However; there is no direct evidence linking melatonin to gut microbiota and AP thus far. To address this gap in knowledge; this study employed a rigorously matched case-control design, which revealed alterations in gut microbial composition in individuals with AP, particularly lower serum melatonin levels in these cases compared to controls. At the level of diversity; the AP case group displayed greater. The present study found that individuals with AP exhibited a significantly altered gut microbiota composition compared to control individuals. The study also revealed that patients with AP exhibited a significantly altered gut microbiota composition compared to control individuals, with a lower Shannon index and higher Simpson index, indicating a lower diversity of microbiota. Additionally, levels of TMAO and LPS were significantly higher in the AP case group compared to the control group, suggestive of an imbalance in gut microbial metabolism. These findings support the growing body of evidence linking gut microbiota dysbiosis to AP development.^[22]^ Similarly, the AUCs for Simpson index and F/B ratio were significantly higher than those for other biomarkers, indicating that these gut microbiota markers may also be useful for AP prediction. This finding is consistent with a previous study by Liu et al (2023), which reported that patients with SAP had a significantly lower Shannon index and higher Simpson index compared to healthy controls.^[21]^ The gut microbiota may contribute to the development of AP by producing toxic metabolites, such as TMAO and LPS, which can lead to inflammation and oxidative stress.^[23]^ Further studies are needed to elucidate the underlying mechanisms and potential therapeutic implications of melatonin and gut microbiota in the context of AP. In addition to its direct effects on the immune system and oxidative stress, melatonin may also play a role in AP by modulating the gut microbiota.^[8,10,21]^ The gut microbiota is a complex community of microbes that inhabits the intestines, and it plays a role in many aspects of health and disease. Recent studies have shown that the gut microbiota is altered in AP patients, and that this alteration may contribute to disease progression. The gut microbiota plays a crucial role in maintaining gut barrier integrity, modulating immune responses, and metabolizing various compounds. In AP, dysbiosis can lead to increased gut permeability, translocation of bacteria and their products (e.g., lipopolysaccharides, LPS), and activation of pro-inflammatory pathways. The higher levels of gut metabolites such as TMAO and LPS observed in the AP group in this study are indicative of gut barrier dysfunction and microbial dysregulation, which can contribute to the systemic inflammatory response in AP.

Several limitations of the study should be considered. The study was conducted in a small sample size, which may limit the generalizability of the findings. Additionally, the study did not assess the causal relationship between melatonin levels, gut microbiota profile, and AP severity. Furthermore, we were only able to assess amylase levels and it is more informative to also evaluate lipase levels. Future studies with larger sample sizes and more rigorous experimental designs are needed to further validate the findings of this study and explore the potential therapeutic applications of melatonin and gut microbiota modulation in AP.

## 5. Conclusion

In conclusion, our findings indicate that melatonin levels are significantly lower in patients with SAP and exhibit a pronounced decline on days 3 and 5, whereas there is no significant change in melatonin levels at the onset of the disease on day 1. The study’s major contribution lies in the investigation of melatonin levels and gut microbiota profile dynamics at different time points during AP progression. The finding that melatonin levels significantly decrease on days 3 and 5 suggests a potential role of melatonin in the early stages of AP, potentially through its anti-inflammatory and antioxidant actions. The study also provides evidence for the association between gut microbiota dysbiosis and AP progression, and the potential of gut microbiota modulation as a therapeutic target for AP. Furthermore, the study revealed that the Simpson index was a more predictive biomarker for AP than other gut microbiota-related biomarkers. These findings have important implications for the diagnosis and management of AP, and further studies are needed to elucidate the underlying mechanisms and potential therapeutic implications of melatonin and gut microbiota in the context of AP.

## Acknowledgments

The authors are grateful to the participants who took part in the study.

## Author contributions

**Conceptualization:** Chao Li, Yangfen Wen, Yuehong Zeng.

**Data curation:** Chao Li, Yangfen Wen, Dan Yu, Yuehong Zeng.

**Formal analysis:** Chao Li, Dan Yu, Yuehong Zeng.

**Funding acquisition:** Yangfen Wen, Qiwen Tong.

**Investigation:** Qiwen Tong, Yi Peng, Yuehong Zeng.

**Methodology:** Qiwen Tong.

**Software:** Yuehong Zeng.

**Validation:** Yisong Rao.

**Visualization:** Yisong Rao.

**Writing – original draft:** Yuehong Zeng.

**Writing – review & editing:** Yisong Rao, Yuehong Zeng.
